# Repeated Oronasal Exposure to Lipopolysaccharide Induced Mucosal IgA Responses in Periparturient Dairy Cows

**DOI:** 10.1371/journal.pone.0103504

**Published:** 2014-07-25

**Authors:** Summera Iqbal, Qendrim Zebeli, Dominik A. Mansmann, Suzanna M. Dunn, Burim N. Ametaj

**Affiliations:** 1 Department of Agricultural, Food and Nutritional Science, University of Alberta, Edmonton, Canada; 2 Department for Farm Animals and Veterinary Public Health, Vetmeduni Vienna, Vienna, Austria; GI Lab, United States of America

## Abstract

This study investigated the effects of repeated oronasal treatment with lipopolysaccharide (LPS) on the humoral immune responses in saliva, vaginal mucus, and the plasma markers of the acute phase response in periparturient dairy cows. One hundred pregnant Holstein cows were administered either 3 increasing doses of LPS (n = 50) as follows: 1) 0.01 µg/kg body weight (BW) on d −28, 2) 0.05 µg/kg BW on d −25, and −21, and 3) 0.1 µg/kg BW on d −18, and −14, or sterile saline solution (controls; n = 50) oronasally for 3 consecutive wk starting at 28 d before parturition. Intensive sampling was conducted on thirty cows (n = 15/group). Multiple saliva, vaginal mucus and blood samples were collected around parturition and analyzed for total immunoglobulin-(Ig)A, plasma serum amyloid A (SAA), lipopolysaccharide-binding protein (LBP), anti-LPS IgA, IgG, IgM, tumour necrosis factor(TNF)-α, and interleukin(IL)-1. Results regarding total secretory IgA (sIgA) antibodies showed greater concentrations in the saliva and an overall tendency for higher total sIgA in the vaginal mucus of the LPS-treated cows. Treatment had no effect on plasma sIgA, IgG, IgM anti-LPS antibodies, haptoglobin, SAA, LBP, TNF-α, and IL-1. Treatments by time interactions were observed for SAA and IL-1 with lowered concentrations of both variables in the plasma of LPS-treated cows after parturition. Overall, repeated oronasal LPS treatment clearly enhanced total sIgA antibodies in the saliva, stimulated their production in vaginal mucus shortly before calving, and lowered plasma IL-1 around parturition, but showed limited effects on markers of the acute phase response in the plasma in dairy cows around parturition.

## Introduction

The period around parturition is often characterized by major biochemical and immunological alterations in dairy cows, which increase the odds for health problems postpartum [1]–[3]. The decline in the immune status of cows appears to be a gradual process, which reaches its nadir immediately before calving [4]. The exact mechanism(s) behind the lowered immune competence in periparturient dairy cows is not completely understood; however, the endocrinological changes and the increased metabolic stress around parturition are believed to play a role [1], [ 2], [ 4]. On the other hand, the presence of lipopolysaccharide (LPS), a cell-wall component of Gram-negative bacteria (GNB), has also been suggested as a factor playing a role in immunosuppression of transition dairy cows [5].

The LPS is persistently present in the mucosal sites of dairy cows; however, it is released in larger amounts in gastrointestinal tract when cows are switched from a high-forage to a high-grain diet immediately after parturition [6]. Research also has demonstrated that the cell-free LPS in the rumen fluid translocates through rumen and colon tissues and that it is found in the systemic circulation, triggering activation of an acute phase response (APR) [6]–[9]. The study conducted by Bryn et al. [5] demonstrated that LPS induces monocytes to produce prostaglandin E_2_ (PGE_2_) that directly suppress T-cell functions and adaptive immune responses, suggesting a role for LPS in the immunosuppression observed during transition period. Furthermore, free LPS in the uterine lumen during early postpartum also induces PGE_2_ secretion by the uterine endometrium [10]–[12].

Mucosal surfaces comprise the first port of entry of bacterial LPS. Therefore, inducing humoral immunity against LPS in mucosal tissues before cows are exposed to high loads of LPS after parturition might prepare them immunologically to prevent harmful effects of LPS translocation. This type of immunomodulation potentially might increase production of secretory immunoglobulin-(sIg)A, which is the dominant isotype synthesized by the mucosal immune system for neutralization of antigens at mucosal surfaces [13]–[15].

Recently, we primed periparturient dairy cows orally with increasing doses of LPS and observed an enhanced response of anti-LPS IgM antibodies in the plasma and improved overall immunity and metabolic health status [15]–[17]. In addition, a study in rats indicated that oral treatment with LPS provides protection against sepsis by increasing concentrations of anti-LPS IgM antibodies [18]. Petzl et al. [19] showed that intra-mammary priming with LPS conferred protection against experimental *Escherichia coli* mastitis in dairy cows. In another study it was shown that oral and nasal administration of monophosphoryl lipid A induced greater salivary and vaginal IgA responses to the group treated orally [20].

To our best knowledge the effects of repeated oronasal treatment with LPS on innate and humoral immune responses and proinflammatory mediators in transition dairy cows are not known. We hypothesized that repeated oronasal treatment of prepartum dairy cows to increasing doses of LPS might improve their innate and humoral immune responses against LPS in dairy cows. Thus, the objectives of this investigation were to evaluate the innate and adaptive immune responses of transition dairy cow to repeated oronasal administration with LPS during the prepartum period.

## Materials and Methods

### Ethical statement

All experimental procedures were approved by the University of Alberta Animal Care and Use Committee for Livestock and animals were cared for in accordance with the guidelines of the Canadian Council on Animal Care [21].

### Animals, study design, and treatments

One hundred pregnant Holstein dairy cows (60 multiparous and 40 primiparous cows) were randomly assigned to two treatment groups according to parity, body conditions score (BCS), milk production, and disease susceptibility from previous year. The average heifer and cow body weights (BW) were 600±20 and 720±30 kg, respectively. Out of 100 cows, 30 of them were randomly assigned to an intensive sampling (n = 15 per group; [10 multiparous (average number of lactation 2.5) and 5 primiparous]) starting at −28 d before the expected day of parturition.

Cows (n = 50) assigned to the treatment group were orally and nasally administered 2 mL and 1 mL of sterile saline solution, respectively, containing 3 increasing doses of LPS from *Escherichia coli* 0111:B4 (Sigma-Aldrich Canada Ltd., Oakville, ON, Canada) at approximately 0800 as follows: 1) 0.01 µg/kg BW once per wk on d −28 before parturition, 2) 0.05 µg/kg BW twice per wk on d −25, and −21 before parturition, and 3) 0.1 µg/kg BW twice per wk on d −18, and −14 before parturition. Cows (n = 50) allocated to the control group received an oral and a nasal treatment of 2 mL and 1 mL of sterile saline solution, respectively, on the same days as for the LPS treatments. Doses of LPS used were based on a previous research conducted by our team and the clinical responses to those doses [16].

The initial crystalline *E. coli* LPS contained 10 mg of purified LPS, which was dissolved in 10 mL of doubly distilled water, as suggested by the manufacturer (Sigma-Aldrich Canada Ltd., Oakville, ON, Canada), and stored in the refrigerator at 4°C until the time of administration. For oral and nasal administrations of LPS to the animals, the daily dose was dissolved in 2 mL and 1 mL of sterile saline solution, and then introduced into the oral and nasal cavity of the cows, respectively, using a disposable 5 mL plastic syringe (Becton, Dickinson and Company, BD, Franklin Lakes, NJ). Similarly, the same amount of carrier [i.e., 2 mL and 1 mL of sterile saline (Sigma-Aldrich Canada Ltd., Oakville, ON, Canada)] was orally and nasally administered to all cows in the control group. All cows that received all doses were included in the experiment independently of the days to calving, whereas all cows that missed a dose because of calving before the expected day of parturition were excluded from the experiment. The experiment lasted for 8 wks i.e., 28 d before the expected day of parturition and 28 d after calving. Cows were housed in tie stalls (122×201 cm), having free access to water throughout the experiment. Shortly before parturition, cows were transferred to the maternity pens (670×427 cm) and returned to their stalls on the next day of parturition.

All animals were fed the same diet once daily at 0800 in their stalls, and daily ration was offered as total mixed ration (TMR) for ad-libitum intake to allow approximately 5% feed refusals throughout the experiment. All animals were offered the close-up diet starting 3 wk prepartum (27% concentrate on DM basis), and they were gradually switched to the lactation diet (50% concentrate on DM basis) during the first 7 d after parturition. Diets were based on locally grown alfalfa hay and barley silage as forage sources, supplemented with rolled barley and corn grain as energy sources, and canola meal and soybean meal as protein sources, as well as mineral- and vitamin supplements. These diets were formulated and offered to the cows to meet or exceed the energy and nutrient requirements of dry and lactating cows as per National Research Council (NRC) guidelines [22]. Cows were milked twice per day at 0400 and 1600.

### Sample collection

Blood samples were collected before each treatment and before the morning feeding at 0800 from the coccygeal vein on d −28, −25, −21, −14, −7, +7, +14, +21, and +28 before the expected day of parturition for plasma haptoglobin and on d −28, −7, +7, +28 before the expected day of parturition for plasma serum amyloid A (SAA), lipopolysaccharide-binding protein (LBP), IgA, IgG, and IgM anti-LPS antibodies, tumour necrosis factor(TNF)-α, and interleukin-1 (IL-1) 15 min prior to administration of the treatment to the experimental cows. Blood samples of approximately 5–8 mL were collected in glass tubes with no additive for blood coagulation (BD Vacutainers 10 mL; Becton, Dickinson and Company, BD, Franklin Lakes, NJ, USA). After collection, blood samples were put immediately on ice, and centrifuged within 20 min (Rotanta 460R, Hettich Zentrifugan, Tuttlingen, Germany) at 3,000 × *g* and 4°C for 20 min. The plasma was separated and stored at −20°C until analyses. During blood withdrawal, no stress response was observed, as indicated from no agitation or overreaction from cows. Feed intake was recorded daily during the entire experimental period. All disease and medication history was recorded for each cow throughout the entire experimental period.

Saliva samples were collected from the oral cavity of the dairy cows on d −28, −7, +7, and +28 around the expected day of parturition using sterile cotton gauze inserted between the cheek and the lower jaw, along the side of the mouth towards the back teeth until the gauze was soaked with saliva. The head movement of the animal was restrained for collection of saliva using conventional restraining technique (i.e., rope halter or held by a person). After collection, saliva was squeezed from the cotton gauze using 60 mL plastic syringe (Becton, Dickinson and Company, BD, Franklin Lakes, NJ, USA), and then placed into a small sterile container, which was then sealed securely and stored at −86°C until analyses for total IgA. No preservatives or additional material was added to the saliva samples.

Vaginal mucus samples were collected on d −28 and −7 before parturition and on d +7 and +28 after the expected day of parturition using sterile insemination plastic pipettes (Becton, Dickinson and Company, BD, Franklin Lakes, NJ, USA). Before sampling, the vulvar area was thoroughly cleaned with water and then disinfected with 30% (vol/vol) iodine solution (Iosan, WestAgro, Saint Laurent, Canada) prior to sampling. Approximately 1–2 mL of vaginal mucus were collected by inserting the insemination pipette into the vagina and close to the cervix by means of aspiration with a plastic syringe. Samples were placed into 1 mL of saline solution in a plastic tube and transported to the laboratory in a refrigerated box. The tubes were sealed securely and stored at −20°C until analyses for total IgA. Before running assay, the samples were centrifuged (Rotanta 460R, Hettich Zentrifugan, Tuttlingen, Germany) at 1,000 × *g* and 4°C for 20 min.

### Sample analyses

Concentrations of total saliva sIgA were measured using a commercially available bovine ELISA kit (Uscn, Life Sciences Inc., USA). The basic principle involved the quantitative measure of IgA in bovine saliva through a sandwich enzyme immunoassay with a working time of 4 h. The microtiter plate pre-coated with antibodies was provided with the kit, and the standards and samples were added to the appropriate wells with a biotin-conjugated antibody preparation specific for IgA. Then avidin conjugated to the enzyme was added to the appropriate wells. The color developed by the substrate addition and was proportional to the concentrations of sIgA, biotin conjugated antibody, and enzyme conjugated avidin. The absorbance was measured at 450 nm, and the lower detection limit for this assay was 0.78 ng/mL. The inter- and intra-assay CV for all the samples tested for total salivary sIgA was less than 10%.

Concentrations of vaginal mucus total sIgA was measured using a commercially available bovine ELISA kit (Uscn, Life Sciences Inc., USA). The basic principle for *in* *vitro* quantitative measure of IgA in bovine biological fluids involved sandwich enzyme immunoassay with a working time of 4 h. The microtiter plate pre-coated with antibodies specific to the sIgA was provided with the kit, and standards as well as samples were added to appropriate wells and incubated. Then avidin conjugated to an enzyme was added to each microplate well and again incubated. The TMB substrate was added and the color developed was shown up only in those wells, which contain sIgA, biotin conjugated antibody, and enzyme conjugated avidin. The absorbance was measured at 450 nm with a spectrophotometer (Spectramax 190, Molecular Devices Corp., Sunnyvale, CA). The minimum detectable concentration of this assay was 0.78 ng/mL. The inter- and intra-assay CV for all the samples tested for total IgA in vaginal mucus was less than 10%.

Concentrations of anti-LPS core IgA, IgG, and IgM antibodies in the plasma were measured using commercially available ELISA kit EndoCab (HK504, Canton, MA, USA), as described previously [16]. In short, antibodies directed against the core structure of endotoxin (i.e., EndoCab) are cross-reactive against most types of LPS, and are measured using a solid phase ELISA based on the sandwich principle with a working time of 2.5 h. The anti-endotoxin core antibodies present in the sample determined the intensity of the colour developed, and the absorbance was measured at 450 nm with a microplate spectrophotometer (Spectramax 190, Molecular Devices Corp., Sunnyvale, CA). The minimum detection levels of IgA, IgG and IgM EndoCab antibodies were 0.156 AMU/ml, 0.0125 GMU/ml and 0.055 MMU/ml, respectively. The CV for the inter- and intra-assay analysis was less than 10% for all the samples tested.

Concentrations of plasma haptoglobin were measured with an ELISA kit provided by Tridelta Development Ltd. (Greystones C., Wicklow, Ireland), as described previously [16]. All samples were tested in duplicate, and the optical density was measured at 630 nm on a microplate spectrophotometer (Spectramax 190, Molecular Devices Corp., Sunnyvale, CA). The minimum detection limit of the assay was 0.25 ng/ml. Inter- and intra-assay CV was less than 10% for this analysis.

Concentrations of LBP in the plasma were quantified with a commercially available ELISA kit, provided with antibody-coated wells that cross-reacts with bovine LBP (Cell Sciences Inc., Norwood, MA, USA), using the method described previously [16]. Plasma samples were initially diluted 1∶1,000; however the samples with lower optical density values than the range of the standard curve were reassayed according to the instructions of the manufacturer with a lower dilution of 1∶500. All samples were tested in duplicate, and the optical densities were measured at 450 nm on a microplate spectrophotometer (Spectramax 190, Molecular Devices Corporation, Sunnyvale, CA, USA). The concentration of plasma LBP was calculated from a standard curve of the known LBP values in human plasma, and the minimum detection limit was 5 ng/ml. The inter- and intra-assay CV for the LBP analysis was less than 10%.

Concentrations of SAA in the plasma were determined using a commercially available ELISA kit (Tridelta Development Ltd., Greystones Co., Wicklow, Ireland) provided with the microtitre strips, coated with monoclonal antibodies specific for SAA, as described previously [23]. In brief, initially samples were diluted 1∶500; however if some of the samples had optical density values below the range of the standard curve, they were reanalyzed in lower dilutions. The inter- and intra-assay CV for the SAA analysis were less than 10%. All samples were tested in duplicate and the optical density values were read on a microplate spectrophotometer (Spectramax 190, Molecular devices Corporation, CA, USA) at 450 nm. The minimum detection limit of the assay was 18.8 ng/mL.

A commercially-available ELISA kit was used to quantify concentrations of TNF-α in the plasma (Bethyl Laboratories, Inc. TX, USA). Initially, incubation of diluted samples and standards (100 µL) were done in the coated plate for 1 h, followed by washing. Then, the plate was incubated with 100 µL of detection antibody and horseradish peroxidase (HRP) substrate for 1 h and 30 min, respectively, and each of the incubations were followed by washings for 4 times. The detection antibody solution cross-reacts with the antibodies attached to the coated wells. The addition of 100 µL of TMB solution allow the enzymatic reaction, and the colour developed was proportional to the amount of anti-TNF-α antibodies present in the sample. The absorbances were measured at 450 nm with a spectrophotometer (Spectramax 190, Molecular Devices Corp., Sunnyvale, CA). The minimum detection limit of the assay was 0.078 ng/mL. The inter- and intra-assay CV for the analysis of TNF- α was less than 10%.

Concentrations of IL-1 in the plasma were determined by a commercially available bovine ELISA kit (Cusabio biotech Co., Ltd, Newark, USA), based on the competitive inhibition of an enzyme immunoassay technique. According to the manufacturer, antibodies specific to IL-1 were pre-coated onto microplate wells. Standards and samples were then incubated with biotin-conjugated IL-1, which leads to competitive inhibition reaction between IL-1 (standards or samples) and biotin-conjugated IL-1 with the pre-coated antibody specific for IL-1. The avidin conjugated to HRP was added to each microplate well and incubated following the addition of substrate solution to the wells. The color developed was considered opposite to the amount of IL-1 in the sample. Further development of color was stopped by adding stop solution and the intensity of the color was measured at 450 nm with a spectrophotometer (Spectramax 190, Molecular Devices Corp., Sunnyvale, CA). The minimum detection level of bovine IL-1 was <125 pg/mL. The inter- and intra-assay CV for the IL-1 analysis was less than 10%.

### Statistical analysis

The data were analyzed using the MIXED procedure of SAS (version 9.2; SAS Institute Inc.) accounting for the fixed effects of treatment and wk relative to calving, and their two-way interaction. Data are shown as least-squares means (LSM) and standard error of the mean (SEM). Treatment LSM were compared at each time point using the SLICE option of SAS [16]. The measurements taken on the same cow but at different sampling days were considered as repeated measures in the model. The Kronecker product of a completely unrestricted variance-covariance matrix (for administration day) was used to account for repeated measures taken on individual cows across time [17]. The measurements taken on d −28 were considered as covariates in the analysis. The covariance structure of the repeated measurements for each variable was modeled separately according to the lowest values of fit statistics based on the Bayesian information criteria (BIC). The significance limit was declared at *P*<0.05, whereas a biologically relevant tendency was declared at 0.05≤*P*≤0.10.

## Results

### Plasma, saliva, and vaginal mucus immunoglobulins

**Figure 1:**
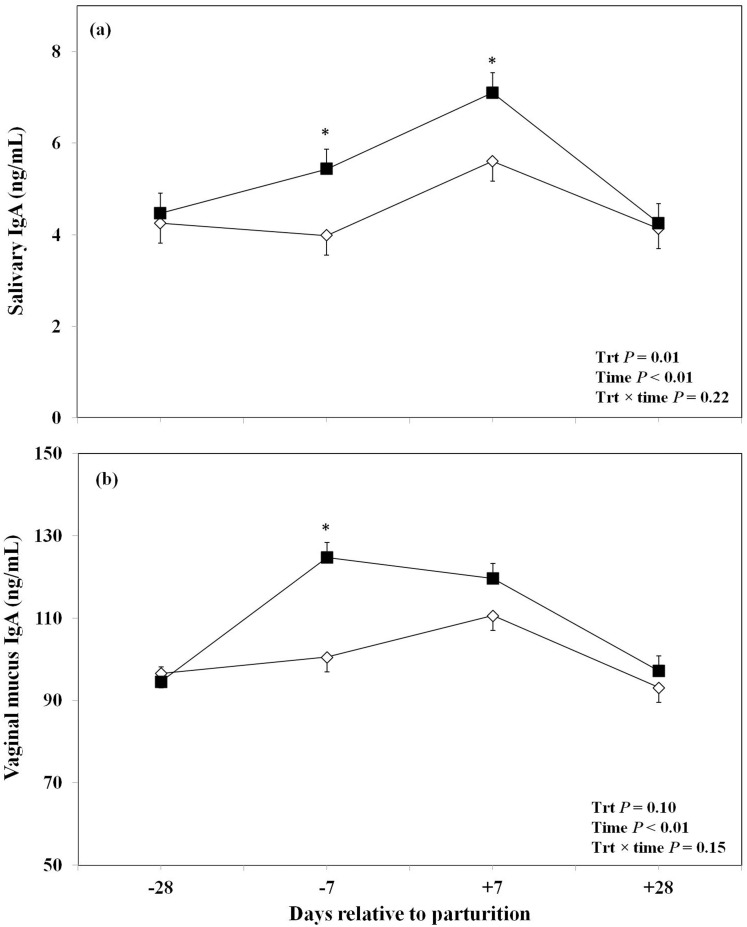
Concentrations of total salivary (a) or vaginal (b) immunoglobulin(Ig) A in periparturient Holstein cows primed oronasally with LPS (▪) or saline (⋄). Cows in the LPS group were administered orally and nasally 2(0.15 M of NaCl), respectively, containing 3 increasing doses of LPS from *E. coli* 0111:B4 as follows: 1) 0.01 µg/kg body weight (BW) on d −28, 2) 0.05 µg/kg BW on d −25, and −21, and 3) 0.1 µg/kg BW on d −18, and −14, whereas control cows received 2 mL oral and 1 mL nasal sterile saline solution. (LSM ± SEM; n = 15; Trt  =  effect of treatment; Time  =  effect of sampling day, Trt×time  =  effect of treatment by sampling day; *indicates treatment differences at specific time points at *P*<0.05).

**Figure 2:**
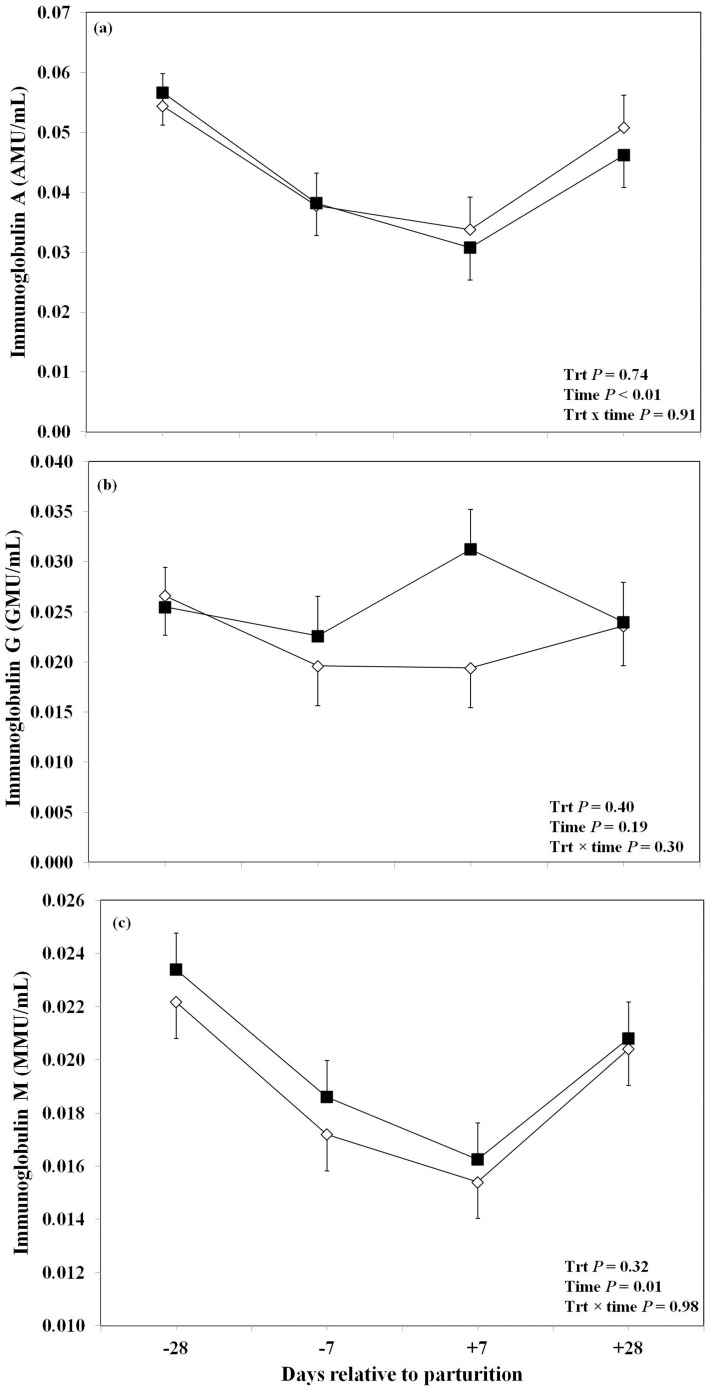
Concentrations of plasma immunoglobulin(Ig)-A (a), IgG (b), and IgM (c) in periparturient Holstein cows primed oronasally with LPS (▪) or saline (⋄). Cows in the LPS group were administered orally and nasally 2(0.15 M of NaCl), respectively, containing 3 increasing doses of LPS from *E. coli* 0111:B4 as follows: 1) 0.01 µg/kg body weight (BW) on d −28, 2) 0.05 µg/kg BW on d −25, and −21, and 3) 0.1 µg/kg BW on d −18, and −14, whereas control cows received 2 mL oral and 1 mL nasal sterile saline solution. (LSM ± SEM; n = 15; Trt  =  effect of treatment; Time  =  effect of sampling day, Trt×time  =  effect of treatment by sampling day).

The effect of oronasal administration of LPS on saliva and vaginal mucus immunoglobulins are shown in Figure 1. We observed a significant overall effect of oronasal LPS treatment on salivary sIgA. The LPS-treated cows had greater overall concentrations of salivary sIgA than the control group. Furthermore, this effect was more pronounced at day −7 prepartum and +7 postpartum with greater concentrations of salivary sIgA in the treatment group than control cows (Figure 1a). The LPS-treated group also showed a tendency to a high overall concentration of vaginal mucus sIgA. We observed that this effect was more significant at day −7 prepartum in LPS-treated cows compared to the control ones (Figure 1b). Data regarding the oronasal effect of LPS on anti-LPS antibodies in plasma are shown in Figure 2. There was no significant effect of LPS administration on IgA anti-LPS antibodies in the plasma (Figure 2a). Although, the concentrations of IgG anti-LPS antibodies in the plasma seemed greater in the LPS-treated cows than saline treated ones at day +7 postpartum the overall effect of treatment did not reach the point of significance (Figure 2b). Moreover, no overall effect or significant differences at various time points were found between the two treatment groups regarding plasma IgM anti-LPS antibodies (Figure 2c).

**Figure 3:**
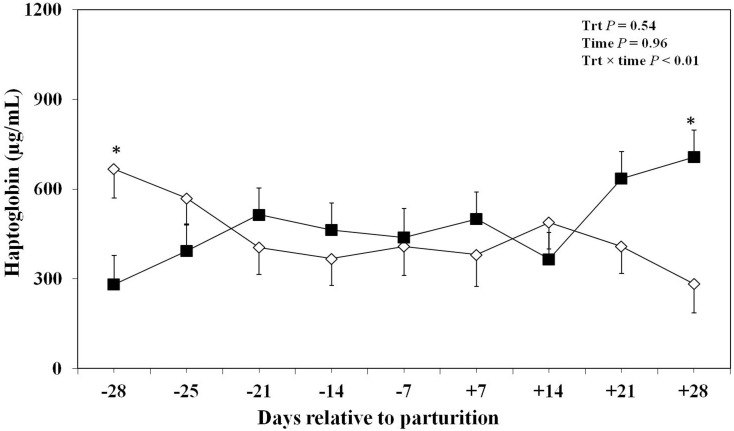
Concentrations of plasma haptoglobin in periparturient Holstein cows primed oronasally with LPS (▪) or saline (⋄). Cows in the LPS group were administered orally and nasally 2(0.15 M of NaCl), respectively, containing 3 increasing doses of LPS from *E. coli* 0111:B4 as follows: 1) 0.01 µg/kg body weight (BW) on d −28, 2) 0.05 µg/kg BW on d −25, and −21, and 3) 0.1 µg/kg BW on d −18, and −14, whereas control cows received 2 mL oral and 1 mL nasal sterile saline solution. (LSM ± SEM; n = 15; Trt  =  effect of treatment; Time  =  effect of sampling day, Trt×time  =  effect of treatment by sampling day; *indicates treatment differences at specific time points at *P*<0.05).

**Figure 4:**
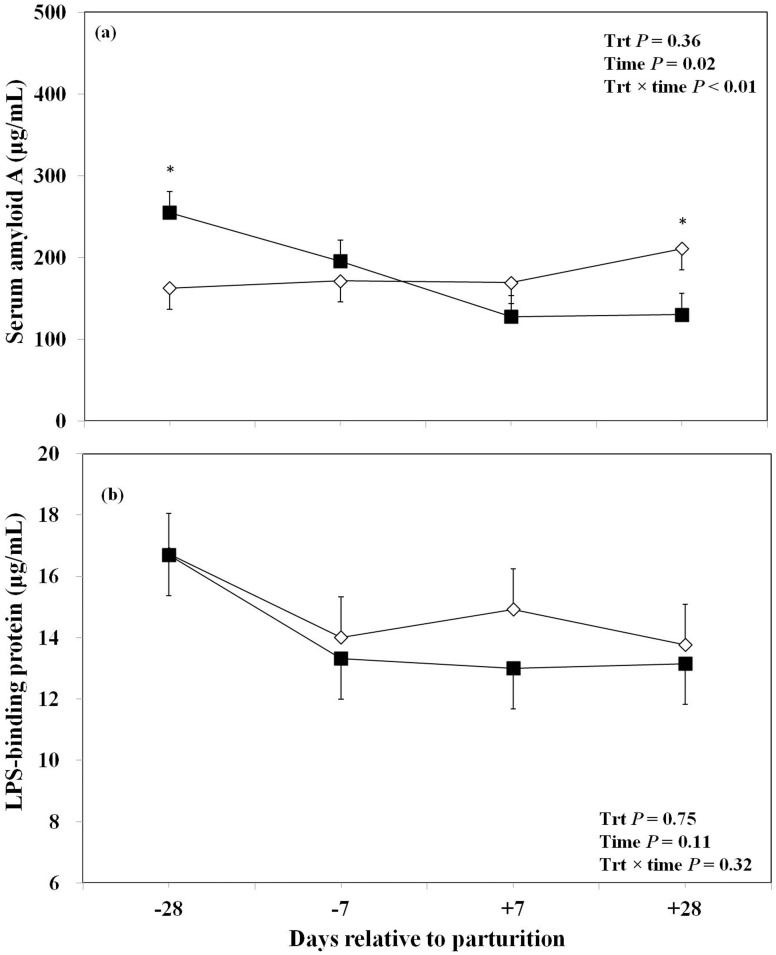
Concentrations of plasma serum amyloid A (a), and lipopolysaccharide binding protein (b), in periparturient Holstein cows primed oronasally with LPS (▪) or saline (⋄). Cows in the LPS group were administered orally and nasally 2(0.15 M of NaCl), respectively, containing 3 increasing doses of LPS from *E. coli* 0111:B4 as follows: 1) 0.01 µg/kg body weight (BW) on d −28, 2) 0.05 µg/kg BW on d −25, and −21, and 3) 0.1 µg/kg BW on d −18, and −14, whereas control cows received 2 mL oral and 1 mL nasal sterile saline solution. (LSM ± SEM; n = 15; Trt  =  effect of treatment; Time  =  effect of sampling day, Trt×time  =  effect of treatment by sampling day; * indicates treatment differences at specific time points at *P*<0.05).

The effect of oronasal LPS exposure on plasma haptoglobin is shown in Figure 3. The overall concentrations of plasma haptoglobin were not significantly different between the LPS treated group and the control group. However, the concentrations of plasma haptoglobin were lower at day −28 prepartum and greater at day +28 postpartum in the LPS-treated cows than control group. Data regarding overall effect of treatment and the effect of LPS challenge at different time points on plasma SAA and LBP are reported in Figure 4. There was no overall significant effect of oronasal LPS administration on plasma SAA. Of note, this effect was significant at day −28 with greater concentrations of plasma SAA in the LPS-treated group, which declined almost 2-fold the wk after parturition, and remained at this level until the end of the experiment (i.e., 254 µg/mL at −28 day versus 130 µg/mL at +28 day around parturition). Concentrations of plasma SAA in the control cows increased from 160 to 210 µg/mL from −28 to +28 day around parturition, respectively (Figure 4a). However, oronasal LPS treatment did not have an overall effect or sampling day effect on plasma LBP in this study (Figure 4b).

**Figure 5:**
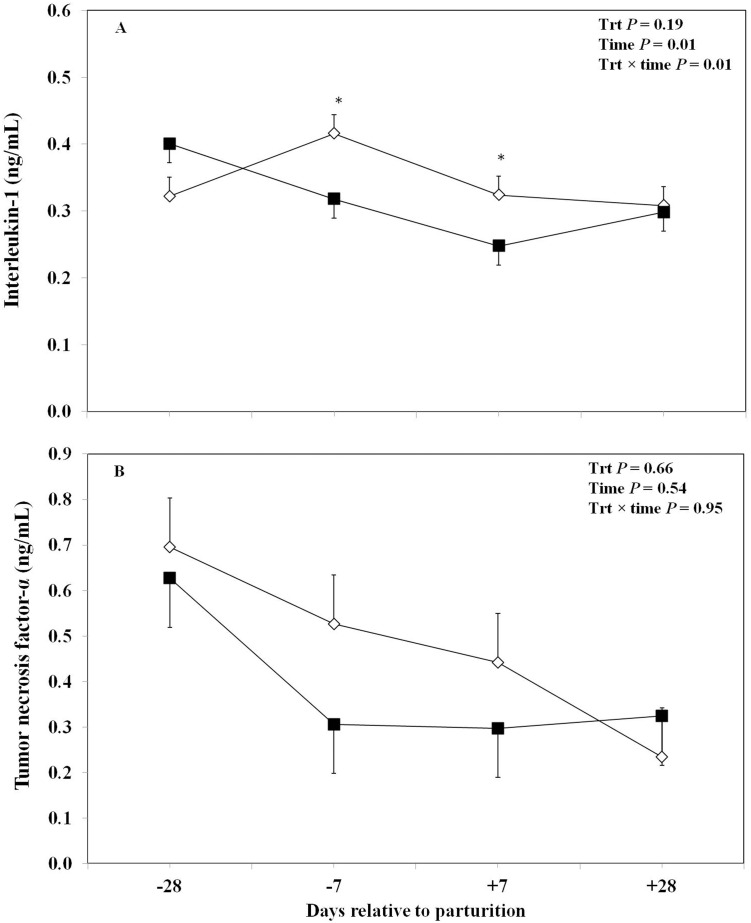
Concentrations of plasma interleukin-1 (a) and tumour necrosis factor-α (b) in periparturient Holstein cows primed oronasally with LPS (▪) or saline (⋄). Cows in the treatment group were administered orally and nasally 2(0.15 M of NaCl), respectively, containing 3 increasing doses of LPS from *E. coli* 0111:B4 as follows: 1) 0.01 µg/kg body weight (BW) on d −28, 2) 0.05 µg/kg BW on d −25, and −21, and 3) 0.1 µg/kg BW on d −18, and −14, whereas control cows received 2 mL oral and 1 mL nasal sterile saline solution. (LSM ± SEM; n = 15; Trt  =  effect of treatment; Time  =  effect of sampling day, Trt×time  =  effect of treatment by sampling day; * indicates treatment differences at specific time points at *P*<0.05).

Results regarding the effects of oronasal LPS on IL-1 and TNF-α are presented in Figure 5. Data showed that LPS treatment did not have a significant influence on the overall concentrations of plasma IL-1. However, plasma IL-1 was significantly lower at day −7 prepartum and +7 days postpartum in the LPS-treated cows than those in the control group. On the other hand, the control cows showed elevated plasma IL-1 from day −28 to −7 before calving, and then there was a slight decrease immediately after parturition at day +7 (Figure 5a). There were no significant overall differences in the plasma concentrations of TNF-α between the treated and the control cows. Also, there was no significant influence of the treatment at any specified time point (Figure 5b).

## Discussion

The present study investigated whether repeated administration of increasing doses of LPS in the oral and nasal mucosa would affect cow’s ability to mount an effective immune response against LPS during the periparturient period. The results of this study demonstrated that oronasal LPS modulated mucosal immunity, but had little effects on the reactants of APR. To the best of our knowledge, this is the first study, which associates the oronasal LPS treatment with mucosal humoral responses in dairy cows.

The most interesting finding of this study was that oronasal LPS increased concentrations of total salivary sIgA antibodies in the treated cows around parturition. Secretory IgA constitutes the largest humoral immune response and serves as the first line of defence predominantly in the epithelial sites through a process known as the immune exclusion [13], [ 14]. In the mucosal sites, the sIgA promotes the clearance of antigens by blocking their access to epithelial receptors, entrapping them in mucus, and facilitating their removal by peristaltic and mucociliary activities [14]. Lipopolysaccharide has been demonstrated to directly activate local B lymphocytes and induce sIgA secretion in a T-cell independent way [24], [ 25]. Our data are in agreement with a previous study that showed that oral immunization with GNB *Brucella melitensis* enhanced salivary anti-LPS IgA responses [26]. The underlying mechanism might be related to priming of local plasma IgA^+^ B cells. The primed lymphocytes enter into the bloodstream and preferentially home to various mucosal surfaces via the common mucosal immune system, and then differentiate into IgA-producing cells at adjacent mucosal layers [27].

Results also showed a tendency for greater concentrations of total sIgA antibodies in the vaginal mucus of cows treated oronasally with LPS. This indicates that oronasal LPS, at the dose used in this study, was able to induce a short-lived IgA response in the vagina of dairy cows. Induction of an immune response in the genital tract of transition cows is of particular importance since uterine infections are very common and cause infertility and are the number one reason for culling of dairy cows. Previous studies demonstrated that intranasal immunization with cholera toxin B subunit alone or mixed with bacterial protein stimulated IgA antibody responses in the vaginal secretions of human volunteers and in rhesus monkeys [28], [ 29]. Besides nasal antigenic stimulation, results from studies in animal and human models have shown that oral immunization with GNB also induces a pronounced immune response in the genital tract and in the vaginal secretions [30], [ 31]. Although oral and nasal mucosae are anatomically separate regions immunization at those sites induces specific sets of mucosal homing receptors during the interaction of T and B cells with antigen, which create functional connectivity and can stimulate effector T and B cell responses at adjacent mucosal tissues such as the intestinal and urogenital tracts [32], [ 33].

Data showed that plasma IgA, IgG, and IgM anti-LPS antibodies were not affected by the oronasal administration of LPS. There was a sharp increase of plasma anti-LPS IgG concentration in the treated cows immediately after parturition. Qadri et al. [34] observed that LPS from *Vibrio Cholera* enhanced IgG antibody responses of all four IgG subclasses in infected patients and that IgG antibodies showed important antibacterial activity and capacity to bind with complement. Interestingly, a new line of investigation indicated the TI memory B cells are established during administration of pure bacterial polysaccharide [35]. Our results suggest that oronasal treatment with LPS may have induced secondary humoral immunity in periparturient dairy cows.

Repeated oral challenge with LPS did not have an overall effect on concentrations of haptoglobin, SAA, and LBP in the plasma; however, cows in the treated group had lower plasma SAA after parturition. It is well established that plasma APP are non-specific immune responses and are produced when liver hepatocytes are stimulated by pro-inflammatory cytokines [36]. A number of studies have indicated that SAA released into the systemic circulation binds, neutralizes, and removes LPS from systemic circulation through liver hepatocytes [6], [37]–[39].

Results also indicated that oronasal treatment with LPS had no effect on plasma TNF-α. Furthermore, treatment did not show an overall effect on plasma IL-1, although it was lower in the treated cows around calving. The lower levels of IL-1 in the treated cows during 2 wk around calving may indicate a lower systemic inflammation in the treated cows during the transition period [36], [ 40], [ 41]. Humblet et al. [42] showed that calving is associated with enhanced concentration of pro-inflammatory cytokines in dairy cows. Interleukin-1 is produced by liver macrophages when they bind to LPS [35]. Furthermore, IL-1 cascade has an early and important role in neutrophilic recruitment in response to LPS challenge at the site of inflammation [43].

In summary, data from this study showed that repeated oronasal treatment with LPS enhanced concentrations of total salivary sIgA and had a tendency for greater total sIgA in the vaginal mucus. There were no effects of the oronasal LPS on plasma IgA, IgM, and IgG anti-LPS antibodies in the treated cows. Results also demonstrated that APP including haptoglobin, SAA, LBP, and TNF-α were not affected by the treatment; however, IL-1 was lowered in the treated cows around calving compared to the control animals. Overall, data indicated that oronasal LPS administration affected mucosal sIgA secretion but had little effect on blood variables related to APR.
